# Supplementary material for: Understanding variation in health service coverage and maternal health outcomes among districts in Rwanda – A qualitative study of local health workers’ perceptions
Source: PLoS One. 2019 Oct 7;14(10):e0223357. doi: 10.1371/journal.pone.0223357 (PMC6779235; doi:10.1371/journal.pone.0223357)
Supplement: S1 File — (PDF) [file pone.0223357.s001.pdf]

FGD/dialogue guide

For the Director of health, officers in-charge of social affairs at sector level, director of hospital, heads of Health centres, nurses working in maternity and Neonatology services at District Hospital, and nurses working in ANC / Maternity.

Introduction

My name is …………………………………………….. working in collaboration with MoH.

The Rwanda Ministry of Health and UMC Radboud University are conducting a study to determine the factors that explain why certain districts perform much better than others, so that these can be addressed in districts with poor performance and further promoted in districts that perform well.

It is our intention to do some in-depth interviews with various officers and groups in the district about maternal health: in particular (a) what goes well, (b) what are the challenges that your are facing, and (c) how you are dealing with these challenges. We are not on a fault-finding mission. We would like you to feel at ease and speak freely about your personal experiences and viewpoints, where possible supported with examples and hard facts. We would like to encourage you not to speak about positive examples only, and the bright side of your work: we are also interested to hear from you what goes less well, and ideas how problems can be solved.

We have identified 4 districts (out of the 30 health districts in Rwanda) to conduct this study. It was not a random choice for us to select your district. We have looked at some of your HMIS and SISCOM statistics for 2013 and 2014; in particular statistics on maternal health. We found a few remarkable things in your statistics, which are worth exploring with you.

You have all been identified to participate in this discussion as key stakeholders in your district. We will also be speaking with health staff – from the hospital, from health centres – as well as with CHW, so that we obtain the different perspectives. That then will allow us to analyse the reasons of your performance, and compare it with other districts. We expect to draw conclusions and identify lessons, which we will of course share with you at some point.

Our discussion shall be tape recorded so as to allow us capture everything that we discuss here today. Please do note that none of you will be linked directly to the findings of this study. Therefore feel free to discuss and dialogue over the subject matter.
During our dialogue, we shall have some guiding questions but you are also free to ask any question that you might have in mind and then we shall dialogue over it.
Everything that we discuss here shall be kept confidential and used only for the purposes of this study. Your participation is voluntary and you are at will to answer or not to answer any question or discussion point at any time. You are also free to exist the discussion at anytime and there will be no negative consequences to you or your family.
Some time back, we undertook an analysis of HMIS indicators from your district and other districts about key reproductive health indicators. In this study, we rated the districts using key indicators that we grouped as coverage and quality indicators as shown in the attached table .

Your district was rated as poor/good performing in the coverage, process and outcome indicators. Today we want to understand this rating further by examining the factors responsible for your performance.

Discussion questions / Topic guide

Icebreaker

0. What are you most proud of in your area of work?
   - Is there any good achievement that you are particularly proud of?
   - Is there any area that you are less proud of?

1. Provide the table that indicates district scores on maternal health.
   a. Ask respondents whether they can confirm that these scores do indicate indeed how the district is doing.
      Are there any indicators of which the scores do not represent the actual situation?
   b. Focus on coverage indicators first.
   c. Then on outcome indicators.
   d. And finally on process indicators.

   Seek confirmation from the respondents and note:
   - Which of the scores/statistics are actually correct – mark them green
   - Which ones are most likely not correct – mark them red, and if possible put the correct score/estimate
   - Which of the scores are they not able to validate.

2. Invite respondents to comment on 2 or 3 scores that are particularly good:
   - What has made it possible to achieve such a good level of performance?
   - What is so typical/special about this district that has made this possible?
   - **Probe to find out whether there is anything unique about the district:**
     - **what is it that this district has / does, which most other districts do not have / do?**

3. Invite respondents to comment on 2 or 3 scores that are below average:
   - Are there any particular reasons why these scores are low?
   - What are the reasons behind this? (PROBE for barriers.)
   - Have you tried to reverse the situation? Have you tried to address the underlying problems? Any special initiatives that you have undertaken?
   - Did that have any effect? Why, or why not?
   - What else could be done? PROBE for opportunities that are within the reach of the district itself; and for other actions that would be required.
   - **Probe to find out whether there is anything unique about the district:**
     - **what is it that it doesn't have / do, contrary to most other districts in Rwanda?**

4. Do you take any special approach towards adolescents (versus adults), when it comes to achieving targets?
   Any special approach towards teenage girls? Towards teenage boys?

Tools that we shall need

1. Flip charts
2. Markers : different colours
3. Recorders, printed copies of the maternal health indicators + district scores.
