# Supplementary material for: Understanding variation in health service coverage and maternal health outcomes among districts in Rwanda – A qualitative study of local health workers’ perceptions
Source: PLoS One. 2019 Oct 7;14(10):e0223357. doi: 10.1371/journal.pone.0223357 (PMC6779235; doi:10.1371/journal.pone.0223357)
Supplement: Attachment — Submitted filename: Response to reviewers.docx [file pone.0223357.s002.docx]

Below I provide a detailed response to the comments from 4 reviewers received on 1 April, 2019. I also provide a version of the revised manuscript with track changes as a supplementary file, as well as a clean version of the revised manuscript.

| **#** | **Comment received** | **Response** |
| --- | --- | --- |
| **Reviewer #1** | | |
| 1 | The manuscript is already published elsewhere,  Bijlmakers L, Sayinzoga F, Tetui M, Van Der Velden K, Van Dillen J. Understanding variation in maternal health service coverage and maternal health outcomes among districts in Rwanda. . 2019 Jan 1:516112. <https://www.biorxiv.org/content/10.1101/516112v1.abstract>  You cannot publish a single research manuscript in two different journals at the same time. | Unfortunately this comment has caused a lot of confusion. We did follow the correct procedures and I’m glad that our appeal against the initial negative decision has been accepted and that we have been given the chance to resubmit. |
| **Reviewer #2** | | |
|  | Thank you very much for the opportunity to review this interesting qualitative study. Overall, this is very well written and a topic of great relevance. However, there are some important considerations that I would like to offer. | - |
| 1 | #1 Title line 1-2 It seems like the data collection method is missing in the title. Please, include this according to journal guidelines. | We have now added the following subtitle: **‘a qualitative study of local health workers’ perceptions’** |
| 2 | #2 Introduction line 71 Please, define upon first appearance in the text. | SDG has now been spelt out. |
| 3 | # 3 Aim line 82-85 Is it even possible to get an in-depth understanding of factors, barriers and enablers from focus group discussions or are you instead interested in different perspectives? Aim and method should be aligned. Furthermore, it will be helpful to specify whose perspectives you take when obtaining an understanding factors affecting maternal health outcomes. | We have rephrased the aim of the study as follows (the parts in bold have been added):  ‘The **aim of the** present study was to obtain **the perspectives of health professionals and community health workers on** factors that **determine health service coverage and maternal health outcomes so as to understand** variations between districts and identify the prevailing barriers and enablers to improve maternal health.’  For consistency purposes, we have also rephrased the objective of the study stated in the abstract:  ‘To **obtain the perspectives of health professionals and community health workers on** factors that determine health service coverage and maternal health outcomes so as to understand variations between districts.’ |
| 4 | # 4 Data collection techniques and 113-130 You mention that sixteen FGD were conducted (four in each district) with different health workers, managers and politicians. However, information on the size and combination of participants in the FDG is lacking. Additionally, information is needed on where and how the focus group and individual key informant interviews were conducted. Furthermore, sampling strategy is not described; including how and why participants are selected and if there were any challenges with recruitment.  Moreover, considerations what the key informant interviews will bring extra to the study are needed. What is the added value?  There are no reflections on data saturation, the role of researchers and interviewers and the relationship between them and the participants. Please, provide these details.  And please describe the content and provide examples of questions of the structured topic list or insert the list as supplementary information. | We have reorganised and improved the Data collection techniques and tools section (under Methods), which was not very clear indeed. We now start this section by explaining who took part in the four FGDs in each of the 4 districts (for a total of 16 FGDs), indicating also the number of participants (range for the 4 districts), and how they were recruited.  As to the key informant interviews, we now also explain why we conducted those (the part in bold below has been added):  ‘In Bugesera district, three additional individual key informant interviews were conducted, **of which the purpose was to complement the information collected through FGDs, particularly on how maternal health services are organised and coordinated in the district:** one interview with …’  We have added: ‘**Discussion and reflection on the emerging themes by the study team during evening meetings on the day the interviews were held allowed decisions on data saturation and identification of topics that needed further exploration during subsequent field work days.’**  Also added: **‘One of the members of the research team present during the interviews is a senior Ministry of Health officer (FS). Apart from bringing in why this study was being conducted, he did not actively take part in the interviews.’**  We have provided more information on the topic list, as follows (the parts in bold have been added):  ‘In order to provide structure to the discussions, a topic list was developed, **containing questions on achievements in maternal health that participants were particularly proud of (or not so proud of) and on particular reasons that explained in their perception the district’s most prominent scores and rankings (presented in Table 2).** This topic list was used in a flexible manner, with ample room to **comment on these scores and ranking and to elaborate on specific actions that had been undertaken to improve maternal health service coverage and health outcomes.** |
| 5 | # 5 Ethical considerations There are not enough details on how the research was explained to participants or on confidentiality. Please, elaborate on these issues. | We have added a paragraph on **Ethical considerations** (in the Methods section, before the Data analysis paragraph). |
| 6 | # 6 Results from line 141 Unfortunately, there are no quotes to support the findings in the beginning of the result section (line 145-172). This section should be elaborated, and more data should be presented.  Furthermore, it is not clear to what extent contradictory data are considered and how the key informant interviews contribute compared to FGD. | We have added two quotes to the first part of the Findings section: see the revised manuscript.  In the Data collection techniques and tools section we now explain why the key informant interviews were held – as follows:  ‘In Bugesera district, three additional individual key informant interviews were conducted, **of which the purpose was to complement the information collected through FGDs, particularly on how maternal health services are organised and coordinated in the district.’** |
| 7 | # 7 Limitations line 353-364 This section is too poorly described; please, elaborate on methodological limitations in this study (e.g.: sampling strategy, language ). | We have added the sampling method as a methodological limitation of the study. We have rephrased the argumentation around the other limitation of not addressing quality of care issues in the interviews). Although the interviews were held in different languages, as explained in the Methods section, this did not form a methodological barrier in our view. |
| 8 | # 8 References The reference list appears relevant and up-to-date. | - |
| **Reviewer #3** | | |
| 1 | There should have been some quantitative data to support the qualitative aspect of the study. Also the focus group discussions should have been each group with people of -demographic characteristics. Not the senior and the junior. | Quantitative data showing the performance of the four districts are provided in Table 2. We have refrained from providing counts of answers provided by the interviewees, as this is not recommended in the literature on quality research (e.g. the standard work of R.K. Yin (1989) Case study research: design and methods.  We do not fully understand the second part of the comment. FGDs were held with homogenous groups, as is recommended in the literature. |
| **Reviewer #4** | | |
|  | Dear PLOS one research editorial group thank you for your consideration our manuscript title ‘Understanding variation in maternal health services coverage and maternal health outcomes among districts in Rwanda”. We addressed of reviewers as follows: | - |
| 0 | General Comments: -The abstract section not as per the standard. Try to make attractive. -The title needs to be self explanatory. -There is of tenses in the whole manuscript.  Specific Comments Section: On the introduction; ¬ The first paragraph is taken only from one reference. Needs thorough revision.  ¬ The knowledge gap is not as strong as expected. ¬ Similarly the statement of the problem.  I have evaluated by Critical Appraisal Skills Programme (CASP) Ten Questions to Help You of Qualitative Research from book of Qualitative Methods in Public Health; A Field Guide for Applied Research written by Priscilla R. Ulin, Elizabeth T. Robinson and Elizabeth E. Tolley, 2005) | -We have made some changes to the abstract.  -We have revised the title, also in the light of a comment by Reviewer #2.  -We have edited the manuscript, ensuring consistency of tenses.  We have included 5 additional references to better support the first paragraph of the Introduction: refs 2,3,4,5 and 6.  We have also added a sentence to the Introduction section, specifying the knowledge gap that this study addresses: see our response to the next comment (Comment #1). |
| 1 | Was there a clear statement of the aims of the research? My review response: No | We have addressed that by inserting the following sentence in the Introduction section (just before the aim of the study):  **‘There is a knowledge gap about the perspectives of local health professionals themselves about the prevailing barriers and enablers to improve maternal health.’** |
| 2 | Is a qualitative methodology appropriate? My review response: Yes | - |
| 3 | Sampling strategy My review response: is appropriate, but the sample size is not justified and reason why they quit at 16 FGD was not stated. | With four districts selected and four candidate focus groups per district, we naturally arrived at 16 FGDs.  The Data collection techniques and tools section has been rewritten, as explained above in our response to Comment #4 by Reviewer #2. |
| 4 | Were the data collected in a way that research issue? My review response: Yes, but lacks clarity on how and why? | Unfortunately we do not understand this comment: which aspects in particular would we need to elaborate on? |
| 5 | Was the data analysis sufficiently rigorous? My review response. | - |
| 6 | Has the relationship between researchers been adequately considered? My review: yes | - |
| 7 | Is there a clear statement of the findings? My review response: Yes | - |
| 8 | Do the researchers indicate links between and their own findings on what the data contain? My review response: Seems partial | In the Discussion section we discuss the findings in the light of existing literature on (WHO) health system building blocks. |
| 9 | Are the findings of this study transferable to population? My review response: Needs thorough comparison in the discussion section. The means is different from the result section. | Our study findings, which are based on field work in just four districts, are not representative for the whole of Rwanda, and we do not claim that anywhere in the paper. This study shows that local health professionals are in a position to explain why their districts perform better or less well than other districts: the factors that they mention can be linked to the Health systems framework, but are so context-specific that they remain hidden in macro level analyses. |
| 10 | How important are these findings practice? My review response: Yes | - |

We hope the above responses and the revisions in the manuscript are satisfactory. We once again thank the reviewers for their comments and suggestions and we look forward to seeing this manuscript go through.

Best wishes,
