# Supplementary material for: Understanding variation in health service coverage and maternal health outcomes among districts in Rwanda – A qualitative study of local health workers’ perceptions
Source: PLoS One. 2019 Oct 7;14(10):e0223357. doi: 10.1371/journal.pone.0223357 (PMC6779235; doi:10.1371/journal.pone.0223357)
Supplement: Peer review history

# Author comment: Author response to previous submission

**DOI:** 10.1371/journal.pone.0223357.r001

29 Jun 2019

Attachment Submitted filename: Response to reviewers.docx Click here for additional data file.

# Editor report: Decision Letter 0

**DOI:** 10.1371/journal.pone.0223357.r002

**Authors:** Kahabi Ganka Isangula

20 Sep 2019

Manuscript: Understanding variation in health service coverage and maternal health outcomes among districts in Rwanda – a qualitative study of local health workers’ perceptions

PONE-D-19-18375

Dear Dr. Bijlmakers,

We are pleased to inform you that your manuscript has been judged scientifically suitable for publication and will be formally accepted for publication once it complies with all outstanding technical requirements.

Within one week, you will receive an e-mail containing information on the amendments required prior to publication. When all required modifications have been addressed, you will receive a formal acceptance letter and your manuscript will proceed to our production department and be scheduled for publication.

Shortly after the formal acceptance letter is sent, an invoice for payment will follow. To ensure an efficient production and billing process, please log <gwmw class="ginger-module-highlighter-mistake-type-1" id="gwmw-15675875375221912797707">into</gwmw> Editorial Manager at https://www.editorialmanager.com/pone/, click the "Update My Information" link at the top of the page, and update your user information. If you have any billing related questions, please contact our Author Billing department directly at authorbilling@plos.org.

If your institution or institutions have a press office, please notify them about your upcoming paper to enable them to help maximize its impact. If they will be preparing press materials for this manuscript, you must inform our press team as soon as possible and no later than 48 hours after receiving the formal acceptance. Your manuscript will remain under strict press embargo until 2 pm Eastern Time on the date of publication. For more information, please contact onepress@plos.org.

With kind regards,

Kahabi Ganka Isangula, MD, MPH, PhD

Academic Editor

PLOS ONE

Journal Requirements:

1. Please include a copy of the interview guide used in the study, in both the original language and English, as Supporting Information, or include a citation if it has been published previously.

2. Please amend your current ethics statement to address the following concerns: 

a) Did participants provide their written or verbal informed consent to participate in this study?

b) If consent was verbal, please explain i) why written consent was not obtained, ii) how you documented participant consent, and iii) whether the ethics committees/IRB approved this consent procedure.

Additional Editor Comments (optional):

Reviewer's comments have been addressed to my satisfaction. 

<gdiv></gdiv>

# Editor report: Acceptance letter

**DOI:** 10.1371/journal.pone.0223357.r003

**Authors:** Kahabi Ganka Isangula

30 Sep 2019

PONE-D-19-18375 

Understanding variation in health service coverage and maternal health outcomes among districts in Rwanda – a qualitative study of local health workers’ perceptions 

Dear Dr. Bijlmakers:

I am pleased to inform you that your manuscript has been deemed suitable for publication in PLOS ONE. Congratulations! Your manuscript is now with our production department. 

If your institution or institutions have a press office, please notify them about your upcoming paper at this point, to enable them to help maximize its impact. If they will be preparing press materials for this manuscript, please inform our press team within the next 48 hours. Your manuscript will remain under strict press embargo until 2 pm Eastern Time on the date of publication. For more information please contact onepress@plos.org.

For any other questions or concerns, please email plosone@plos.org. 

Thank you for submitting your work to PLOS ONE.

With kind regards,

PLOS ONE Editorial Office Staff

on behalf of

Dr. Kahabi Ganka Isangula 

Academic Editor

PLOS ONE
